# Availability and Price Variations of Commonly Used Cardiovascular Medicines at Community and Hospital Pharmacies in Gondar Town, Northwest Ethiopia

**DOI:** 10.1155/2024/6551639

**Published:** 2024-09-17

**Authors:** Liknaw Workie Limenh, Tewodros Ayalew Tessema, Ashenafi Kibret Sendekie, Wudneh Simegn, Wondim Ayenew, Melese Legesse Mitku, Yeniewa Kerie Anagaw, Asmamaw Emagn Kasahun

**Affiliations:** ^1^ Department of Pharmaceutics School of Pharmacy College of Medicine and Health Sciences University of Gondar, Gondar, Ethiopia; ^2^ Department of Clinical Pharmacy School of Pharmacy College of Medicine and Health Sciences University of Gondar, Gondar, Ethiopia; ^3^ Department of Social and Administrative Pharmacy School of Pharmacy College of Medicine and Health Sciences University of Gondar, Gondar, Ethiopia; ^4^ Department of Pharmaceutical Chemistry School of Pharmacy College of Medicine and Health Sciences University of Gondar, Gondar, Ethiopia

**Keywords:** availability, cardiovascular disease drugs, community pharmacies, hospital pharmacies, price variation

## Abstract

**Background:** Access to cardiovascular medications is severely hampered by their unavailability and high cost, particularly for the poorest households in developing nations. The availability and price range of cardiovascular medications are significantly limited in both hospital and community pharmacies.

**Objectives:** The aim of this study is to assess the availability and price variations of commonly used cardiovascular medicines in hospital pharmacies in Gondar Town, northwest Ethiopia.

**Methods:** From July 13 to August 6, 2022, a mixed cross-sectional and simulated client survey was carried out at two hospital and 13 community pharmacies in Gondar Town. The analysis and data entry were performed using SPSS Version 25 and EpiData Version 4.2, respectively. The availability and pricing variations of the medications are given as percentages. The significance was examined using paired *t* tests.

**Results:** On average, community retail pharmacies offered 33.22% of CVD drugs. Aspirin (81 mg), amlodipine (5 mg), atorvastatin (20 mg), and hydrochlorothiazide (25 mg) were the most readily available drugs in community pharmacies. Overall, 28.00% of the hospital pharmacies had available CVD medicines during the course of our analysis. The average cost for the 25 CVD medications in hospital pharmacies was $0.699, with a standard deviation (SD) of 1.513, which was less than the cost at community pharmacies ($2.741 with an SD of 6.015) (*p* = 0.045).

**Conclusion:** CVD medications were more available in community pharmacies than in hospital pharmacies, although there were fewer CVD medications available than recommended by the WHO/HAI (80%) in both hospital and community pharmacies. There was a statistically significant difference between the two prices. Compared to that at hospital pharmacies, the mean price at community pharmacies was greater.

## 1. Introduction

Heart and blood vessel illnesses, referred to as cardiovascular diseases (CVDs), include coronary heart disease, cerebrovascular disease, peripheral arterial disease, congenital heart disease, deep vein thrombosis, and pulmonary embolism [1]. By addressing behavioral risk factors such as cigarette use, poor diet and obesity, physical inactivity, and excessive alcohol use, the majority of CVDs can be prevented [2]. An estimated 100 million people in low- and middle-income countries (LMICs) experience poverty each year as a result of healthcare costs [3]. It is anticipated that this will worsen as the prevalence of noncommunicable diseases increases, endangering the advancements in living standards established over the previous century [4, 5].

The cost of pharmaceuticals significantly affects the affordability of treatment because they make up the majority of treatment costs in LMICs [6, 7]. Although researchers have investigated the cost-effectiveness of specific medications in LMICs, they have not examined the financial burden of several medications that are routinely prescribed to treat chronic conditions such as CVD [8, 9].

The most recent drugs should be available to patients with CVD. Basic drugs that are accessible include aspirin, beta-blockers, angiotensin-converting enzyme inhibitors, and statins [9, 10]. The World Health Organization (WHO) asserts that noncompliance with medical advice is a widespread issue that has negative effects on health and significant financial costs due to time and money lost and untreated illness [11]. By expanding affordable preventative and therapeutic alternatives through prepayment finance contracts, the cost of treating CVD may be significantly reduced [12]. A 2005 World Bank survey revealed that up to 60% of Ethiopian household health expenditures involved pharmaceutical products. Unreliable supply networks and weak financial safeguards are some of the issues encountered. Due to a lack of essential pharmaceuticals in hospital facilities, even individuals with the right-to-fee waivers are forced to purchase from expensive community sources [13].

In Ethiopia, those who are poor are required to pay for their own healthcare, including their own medications, which have important unrecognized economic, psychological, and physical repercussions [14]. With no overt government price controls, pharmaceutical prices in Ethiopia are mostly determined by free market principles [15].

In comparison to other African countries, Ethiopia has lower prices for generic and innovator brand products, according to a 2004 World Health Organization/Health Action International (WHO/HAI) pricing survey. Although the median prices of the cheapest generic equivalents in hospital pharmacies and community pharmacies were 35% and 125% higher than the international reference prices, respectively, it should be noted that hospital procurement prices for the most widely used and least expensive generic products were 29% and 39% lower than the international reference prices [16].

The cost of pharmaceuticals significantly affects the affordability of treatment because they make up the majority of treatment costs in LMICs [5]. A significant portion of the population may face a large financial burden due to CVD medication expenditures [17]. As a result, it is crucial to provide long-term CVD patients with access to and use high-quality, inexpensive medications when necessary [18].

By examining the costs, accessibility, and affordability of these medications, one can establish the degree of access to them. The availability and cost of all necessary CVD medications in Ethiopia, whether in the hospital or community pharmacies, have not been adequately investigated. Understanding the cost of CVD medications in various situations is crucial because the expense of CVD is projected to increase further. The purpose of this study was to compare the prices and availability of regularly prescribed cardiovascular medicines in both community and hospital pharmacies.

## 2. Methods

### 2.1. Study Area

Gondar Town is situated in Amhara Regional State's North Gondar Administrative Zone, 750 km to the northwest of Addis Ababa and 185 km from Bahir Dar. The Gondar population is projected to be approximately 227,100, according to the data from the 2007 National Census. Approximately 500,000 individuals in the city of Gondar are served by 78 community pharmacies and two hospitals, the University of Gondar Comprehensive Specialized Hospital (UoGCSH) and Hayira General Hospital.

### 2.2. Study Design and Period

To determine the availability and price variance of CVD medications in hospital and community pharmacies, a cross-sectional study design combined with the simulated patient (SP) method was used in February 2023.

### 2.3. Sample Size and Sampling Technique (Procedure)

Thirteen community pharmacies in Gondar Town, as well as two hospital pharmacies, participated in the study. Two hospital pharmacies (UoGCSH and Hayira General Hospital) were found in Gondar Town and were chosen for the investigation. Thirteen community pharmacies were selected from the 78 community pharmacies in Gondar Town. Randomly, the pharmacies nearest to the primary hospital facilities were chosen. Within 1 km of the primary hospital facility medication outlet, all community pharmacies were chosen. We chose to utilize these thirteen community pharmacies as a model for this study because they were adjacent to the government pharmacy where we were performing our research. The inclusion of community pharmacies was based on the assumption that they offered the greatest possibility of obtaining medicines not found at the health facility outlets. Additionally, these community pharmacies have a sufficient supply of medications and space for a large number of customers, making it much simpler to locate crucial information for our study.

### 2.4. Selection of Medicines

Twenty-five CVD medicines were selected from the *WHO Model List of Essential Medicines*, *22nd List, 2021*. These included acetylsalicylic acid, clopidogrel, alteplase, amiodarone, amlodipine, bisoprolol, glyceryl trinitrate, digoxin, dopamine, enalapril, epinephrine, furosemide, hydrochlorothiazide, isosorbide dinitrate, lidocaine, lisinopril + amlodipine, lisinopril + hydrochlorothiazide, losartan, methyldopa, sodium nitroprusside, streptokinase, simvastatin telmisartan + hydrochlorothiazide, telmisartan + amlodipine, and verapamil [19]. Due to their importance in comparing statistics on worldwide access to medicines, all available medicines were included. The WHO/HAI procedure guidance for the selection of medications for a survey is in line with this choice [20]. The selection of medications was meant to cover all of the therapeutic agents' various pharmacological classes. Angiotensin-converting enzyme inhibitors, angiotensin receptor blockers, beta-blockers, centrally acting medications, and cardiac medications are among them [21, 22].

### 2.5. Data Collection

After evaluating published literature, a standardized data collection checklist was developed and utilized to collect information about the CVD medications provided in hospital and community retail pharmacies [19, 21, 22]. In hospital pharmacies, the availability of CVD medications was determined by taking the lists of available medications from the medicine store areas; however, in community pharmacies, the investigators asked the pharmacy staff members on-site to provide information about the current inventory of CVD medicines. During that time, the prices of generic versions of comparable medications were obtained and documented from hospital pharmacies, and SP techniques were used to obtain prices from community pharmacies. All of the available CVD medications were listed on the medication list. The investigation covered CVD medicines sold in hospital and community pharmacies in both solid and liquid dose forms. When there were two brands of the same medication, the study used the brand that had recently sold the most. Surrogate consumers were used in place of actual customers to obtain exact retail prices for medications because invoicing is an erratic practice in community pharmacies in Ethiopia. On separate days, the unit retail price of identical medications from the same manufacturer and dose were obtained from hospital and community pharmacies.

### 2.6. Data Quality Control

Prior to the primary data collection in randomly chosen pharmacies, a pretest on the data collection checklist was carried out to guarantee data quality. A suitable modification was made to the data gathering checklist after the pretest. The study did not take into account the pretest. Additionally, the primary researchers supervised the data collection throughout the procedure.

### 2.7. Data Analysis

Twenty-five drug prices that are frequently seen in hospital and community retail pharmacies were calculated. EpiData Version 4.2 and SPSS Version 25 were used for data entry and analysis, respectively. Based on observation of the study medications on the day of the visit, regardless of the amount of previous stock, point-in-time estimates of medication availability were collected from each of the 15 research locations. For hospital and community pharmacies, the availability of a certain medication was measured as the proportion of pharmacy outlets where it was available on the survey day. The formula for calculating percent availability was [23]

 $$\begin{array}{c} \text{Percent availability of medicine}=\frac{\text{Pharmacy outlet having the medicines}}{\text{Number of pharmacy outlets inspected}}\times 100 \end{array}$$

Availability was categorized as *not available* (0%), *very low* (< 30%), *low* (30–49%), *fairly high* (50–80%), or *high* (> 80%) [21].

The difference between the price of medications in hospital pharmacies and that in community pharmacies was divided by the price of medications in hospital pharmacies (given as a percentage) to determine price variation.

 $$\begin{array}{c} \text{Price variation}=\frac{\text{Price of medicine in community pharmacy}-\text{Price of medicine in hospital pharmacy}}{\text{Price of medicine in hospital pharmacy}}\times 100 \end{array}$$

The information is displayed as a percentage along with the mean and standard deviation (SD). Using 95% confidence intervals (CIs), the costs of drugs at hospital pharmacies and privately owned retail pharmacies were compared. The unit price, which is the cost per tablet or vial, was used to calculate each medicine's cost. Using the exchange rate in effect on the first day of data collection ($1 = Br 53.802, February 2023), the median price and the mean price of each drug were also determined. The significance of the difference between the cost of medications in hospital pharmacies and community pharmacies was examined using paired *t* tests.

### 2.8. Operational Definitions

*Generic medicines* are medicines that are offered only after patents or other exclusive rights have been released and do not require a production license from the firm that invented them.

*Brand medicines* are medicines wherein the pioneer or innovator receives a patent and exclusive protection, preventing immediate competition from generic products.

*Hospital pharmacy* is a pharmacy that the government actively regulates.

*Community pharmacy* is an independent pharmacy that is not owned by a hospital-affiliated firm and is not directly connected to any other pharmacy chain.

## 3. Results

### 3.1. Availability of Cardiovascular Medicines in Community and Hospital Pharmacies

A minimum of 0% and a maximum of 69.2% of generic CVD medications were available in community pharmacies. Overall, 33.22% of CVD medications were available in community retail pharmacies on average, with a SD of 25.85%. The most readily available medications in community pharmacies were aspirin (81 mg), amlodipine (5 mg), simvastatin (20 mg), and hydrochlorothiazide (25 mg); these medications were available in nine community pharmacies. The second-highest availability was for 40 mg verapamil and 40 mg furosemide, which are available at eight community pharmacies. However, none of the community pharmacies had 75 mg of clopidogrel, 20 mg of alteplase in a vial, 40 mg/mL of dopamine HCl, or 40 mg of telmisartan + 12.5 mg of hydrochlorothiazide.

During the course of our investigation, hospital pharmacies had a 28.00% overall availability of CVD medications and an SD of 35.59. Most drugs available in hospital pharmacies are generic, which typically cost less than branded drugs. Only four CVD medications were available during the study at two hospital pharmacies: aspirin (81 mg), simvastatin (20 mg), furosemide (40 mg), and methyldopa (250 mg). However, 75 mg of clopidogrel, 20 mg of alteplase in vials, 100 mg of amiodarone, 5 mg of amlodipine, 5 mg of bisoprolol, 40 mg of dopamine HCl, 500 *μ*g of glycerol trinitrate, 5 mg of isosorbide dinitrate, 20 mg of lisinopril + 25 mg of hydrochlorothiazide, 25 mg of losartan, 1.5 million IU of streptokinase in vials, 40 mg of telmisartan + 12.5 mg of hydrochlorothiazide, and 80 mg of telmisartan + 5 mg of amlodipine were not available. The overall mean availability of CVD medicines in both hospital and community pharmacies was 30.63, with a SD of 27.72. Aspirin (81 mg) and furosemide (40 mg) had the highest overall availability, at 84.6% and 80.8%, respectively (Table 1).

### 3.2. Cardiovascular Medicine Price Variation Between Hospital and Community Pharmacies

The mean price of the 25 CVD medicines in hospital pharmacies was $0.699, with an SD of 1.513, which was lower than that in community pharmacies ($2.741, with an SD of 6.015) (Table 2).

Moreover, 40 mg verapamil had the greatest price variation of 1383.27%, followed by 5 mg amlodipine (1332.79%), 50 mg sodium nitroprusside in the ampoule (1089.47%), and 75 mg clopidogrel (983.93%). The lowest price-negative variation was recorded for 1.5 million IU of streptokinase in a vial (38.83%) and 100 mg of amiodarone (82.48%) (Table 3).

### 3.3. Statistical Analysis With Paired-Sample *t* Tests

The calculated *t* value had a degree of freedom of 24, but the tabulated *t* value had a 95% CI of 2.120. There was a statistically significant difference between the two means, as indicated by the *p* value of 0.05. Compared to that at hospital pharmacies, the mean price at community pharmacies was greater (Table 4).

## 4. Discussion

The total availability of CVD drugs was low in both hospital and community pharmacies (28.00% vs. 33.22%), which showed that availability did not meet the WHO/HAI reference's suggested standard of 80% in either setting [24]. This draws attention to a shortage of necessary drugs that may have an effect on patient treatment and health outcomes. The results highlight how crucial it is to set up a mechanism for routinely monitoring and reporting on the cost and availability of CVD drugs. These results were in line with a study conducted in Pakistan, which revealed that none of the drugs under examination had an optimal availability of 80% in both the hospital and community pharmacies [25]. Additionally, this result was consistent with a study conducted in Nigeria that revealed that less than 80% of the surveyed drugs were available across all pharmacies [22]. Aspirin (81 mg), amlodipine (5 mg), atorvastatin (20 mg), and hydrochlorothiazide (25 mg) were the drugs that were the most easily accessible in community pharmacies; these drugs were offered in nine community pharmacies. Only four CVD medications—aspirin (81 mg), atorvastatin (40 mg), furosemide (40 mg), and methyldopa (250 mg)—were available at two hospital pharmacies during the study. Aspirin (81 mg) and furosemide (40 mg) had the highest overall availability in both hospital and community pharmacies, at 84.6% and 80.8%, respectively.

The unavailability of medicines in Ethiopian hospital pharmacies is a consequence of both national and global issues. National challenges include misalignment between procurement lists and guidelines, supply chain deficiencies, and inadequate health insurance coverage [26, 27]. Globally, increased demand and regulatory challenges contribute to shortages [28]. Addressing these issues requires a comprehensive approach that involves improving supply management, fostering transparency, and ensuring that national policies are aligned with healthcare needs [27, 28]. Regulations and policies must be strengthened to drug shortages and ensure steady access to essential medicines [26, 29]. The average availability of CVD medicines was greater in community pharmacies than in hospitals. This outcome is consistent with past research, which revealed that the availability of medications in community pharmacies was greater than that in hospital pharmacies [30, 31]. According to a study conducted in Pakistan, where the mean availability of CVD medications at hospital pharmacies was found to be 27.9%, our findings are in line with that study. Contrary to our findings (which revealed only 33.22% mean availability), Pakistan community pharmacies had higher mean availability (44.8) [25]. In hospital and community pharmacies, the overall mean availability of CVD medications was 30.63%, which was lower than the 33% mean availability reported in Cameroon [21].

The results of the current investigation demonstrated that there were significant (*p* = 0.045) price differences between CVD medications in hospital and community pharmacies in the town of Gondar. In comparison to those at hospital pharmacies, several medications are significantly more expensive at community pharmacies. Although patients would have easier access to necessary drugs in community pharmacies, their affordability was an issue because the mean cost of CVD drugs is higher in community pharmacies, which could impact patient adherence and health outcomes. Similar results to those of the current study were found in a similar study carried out in Ethiopia in 2017 [30], which reported that several prescriptions cost more in community pharmacies than in hospital pharmacies. Additionally, this was consistent with a study conducted in Cameroon that found that the cost of medications was highest in community pharmacies and lowest in hospital facility outlets [21]. The two medicines with the greatest price variations were verapamil 40 mg (1383.27%) and amlodipine 5 mg (1332.79%). In a study conducted in Nepal, 5 mg amlodipine also demonstrated the largest price variation of 667% [32].

Different factors affect medicine pricing variations. A study done in Tanzania that showed retail pharmacies in control programs in Africa suggests that regulatory interventions should include the complex global pharmaceutical that influences local operations. This implies that international market and local regulatory frameworks affect drug pricing [33]. A study in Nepal suggests that lack of regulatory enforcement pricing can lead to price variations in public and private hospitals [28]. The pricing variations demonstrated that consumers spend substantially different amounts depending on which pharmacy they use and where they receive their prescriptions. Increased drug prices can be problematic since they promote nonadherence to medication owing to cost, which is harmful to one's health [34]. According to past studies, price discrepancies are widespread in developing countries, and the community pharmacies are insufficiently regulated [35]. Prices at hospitals and community pharmacies have reportedly varied in a number of countries, including India, Sri Lanka, Pakistan, the Philippines, Malaysia, Peru, Armenia, and Kenya [30, 36–38]. The differing procurement methods employed by hospital and community pharmacies are mostly the reason for the pricing variance. In the first case, purchases are made directly from pharmaceutical companies, while a multilayered procurement structure is used in the second case. Customers pay a high price as a result of each stage's application of a certain markup [30, 36]. The ineffective implementation of the policies, rules, and regulations that now govern community retail pharmacies may also be a factor in price fluctuations. Because the overall population's CVD morbidity has recently increased, the government should act quickly to keep the cost of CVD medicines manageable. The layers between traders and producers should be removed, so that community pharmacies can obtain products directly from manufacturers and reduce the prices shared between them.

### 4.1. Limitations

This study is one of the few of its kind to examine the availability and pricing variations of several CVD medications in both hospital and community pharmacies. The fact that availability was dependent on the presence of a particular medicine in the pharmacy at the time of the survey is one of the study's weaknesses. It is likely that the study's time period saw a sudden shortage of the treatments, so this is not a true reflection of their availability. Second, the poll did not include pharmacies, which are expected to be crucial in the distribution of medications. The study may also be more applicable at the country level if it is conducted using large amounts of data at the regional or national level.

## 5. Conclusion

CVD medications were more available in community retail pharmacies than in hospital pharmacies. The most readily available medications in community pharmacies were aspirin (81 mg), amlodipine (5 mg), atorvastatin (20 mg), and hydrochlorothiazide (25 mg); these medications were available in nine community pharmacies. However, aspirin (81 mg), atorvastatin (40 mg), furosemide (40 mg), and methyldopa (250 mg) were highly available in hospital pharmacies. The mean price was highest for 40 mg verapamil, followed by 5 mg amlodipine. There was a statistically significant difference between the two prices. Compared to that at hospital pharmacies, the mean price at community pharmacies was greater. According to the current investigation, there were fewer CVD medications available than those recommended by the WHO/HAI in both hospital and community pharmacies. Therefore, creating a system to track and report on the cost and availability of medicines on a regular basis is crucial. To ensure that CVD medications are available and priced reasonably, tightening the control system and conducting frequent inspections of community pharmacies may be crucial. The availability and cost discrepancies between CVD medications in large representative samples of hospitals and community pharmacies in Gondar Town require further study.

## Figures and Tables

**Table 1:** Availability of cardiovascular medicines in hospital and community pharmacies.

**Medicine list**	**Hospital pharmacies (%)**	**Community pharmacies (%)**	**Total (%)**
Aspirin 81 mg	100	69.2	84.6
Clopidogrel 75 mg	0	0	0
Alteplase 20 mg in vial	0	0	0
Amiodarone 100 mg	0	23.1	11.6
Amlodipine 5 mg	0	69.2	34.6
Simvastatin 20 mg	50	69.2	59.6
Bisoprolol 5 mg	0	53.8	26.9
Digoxin 0.25 mg	50	23.1	36.6
Dopamine HCl 40 mg/mL	0	0	0
Enalapril 10 mg	50	46.1	48.1
Epinephrine 1 mg	50	38.5	44.3
Furosemide 40 mg	100	61.53	80.8
Glyceryl trinitrate 500 *μ*g	0	15.4	7.7
Hydrochlorothiazide 25 mg	50	69.2	59.6
Isosorbide dinitrate 5 mg	0	7.7	3.9
Lidocaine 20 mg/mL	50	46.1	48.1
Lisinopril 10 mg + amlodipine 5 mg	0	7.7	3.85
Lisinopril 20 mg + hydrochlorothiazide 25 mg	0	15.4	7.7
Losartan 25 mg	0	53.8	26.9
Methyldopa 250 mg	100	53.8	76.9
Sodium nitroprusside 50 mg in ampoule	50	15.4	32.7
Streptokinase 1.5 million IU in vial	0	7.7	3.85
Telmisartan 40 mg + hydrochlorothiazide 12.5 mg	0	0	0
Telmisartan 80 mg + amlodipine 5 mg	0	23.1	11.6
Verapamil 40 mg	50	61.53	55.8

**Table 2:** The mean price of tablets in hospital and community pharmacies.

	**Hospital pharmacy**	**Community pharmacy**
Number of products	25	25
Median	$0.039	$0.244
Interquartile range	0.006–6.663	0.057–21.468
Mean	0.699	2.741
SD	1.513	6.015

**Table 3:** Price variation at hospital and community pharmacies.

**Name of the drugs**	**Price in hospital (unit price in $)**	**Price in retailers (unit price in $)**	**Variation (%)**
Clopidogrel 75 mg	0.006	0.065	983.93
Alteplase 20 mg in vial	0.249	0.497	99.89
Amiodarone 100 mg	0.298	0.544	82.48
Amlodipine 5 mg	0.020	0.286	1332.79
Aspirin 81 mg	0.010	0.074	638.41
Simvastatin 20 mg	0.173	0.681	293.37
Bisoprolol 5 mg	0.016	0.010	542.86
Digoxin 0.25 mg	0.037	0.17663	381.98
Dopamine HCl 40 mg/mL	0.333	0.698	109.37
Enalapril malate 10 mg	0.025	0.074	197.01
Epinephrine 1 mg	1.583	4.943	212.34
Furosemide 40 mg	0.008	0.185	2226.18
Glyceryl trinitrate 500 *μ*g	6.663	17.768	166.67
Hydrochlorothiazide 25 mg	0.009	0.062	600.12
Isosorbide dinitrate 5 mg	2.359	21.468	810.17
Lidocaine 20 mg/mL	2.935	15.547	429.73
Lisinopril 20 mg + hydrochlorothiazide 25 mg	0.057	0.167	194.10
Lisinopril 10 mg + amlodipine 5 mg	0.044	0.181	307.53
Losartan 25 mg	0.131	0.478	264.41
Methyldopa 250 mg	0.025	0.242	860.06
Sodium nitroprusside 50 mg in ampoule	0.021	0.244	1089.47
Streptokinase 1.5 million IU in vial	2.399	3.33	38.83
Telmisartan 40 mg + hydrochlorothiazide 12.5 mg	0.009	0.057	554.04
Telmisartan 80 mg + amlodipine 5 mg	0.039	0.178	359.07
Verapamil 40 mg	0.032	0.478	1383.27

**Table 4:** Test of significance for price variation.

**Comparison**	**Mean difference**	**95% confidence interval of the difference**		**t** ** value**	**p** ** value**
Price in community vs. price in hospital pharmacies	2.042	0.054	4.030	2.120	0.045

## Data Availability

The datasets used and/or analyzed in this study are included as additional files, but additional information can be made available by the corresponding author upon request.

## Nomenclature

CI: confidence interval
CVD: cardiovascular diseases
HAI: Health Action International
LMICs: low- and middle-income countries
SD: standard deviation
SP: simulated patient
UoG: University of Gondar
UoGCSH: University of Gondar Comprehensive Specialized Hospital
WHO: World Health Organization
